# Performance Index for Extrinsic Calibration of LiDAR and Motion Sensor for Mapping and Localization

**DOI:** 10.3390/s22010106

**Published:** 2021-12-24

**Authors:** Gamin Kim

**Affiliations:** Department of Smart Vehicle Engineering, Konkuk University, Seoul 05029, Korea; yondoo20@gmail.com

**Keywords:** LiDAR, extrinsic calibration, performance index, motion sensor, localization, mapping

## Abstract

Light Detection and Ranging (LiDAR) is a sensor that uses a laser to represent the surrounding environment in three-dimensional information. Thanks to the development of LiDAR, LiDAR-based applications are being actively used in autonomous vehicles. In order to effectively use the information coming from LiDAR, extrinsic calibration which finds the translation and the rotation relationship between LiDAR coordinate and vehicle coordinate is essential. Therefore, many studies on LiDAR extrinsic calibration are steadily in progress. The performance index (PI) of the calibration parameter is a value that quantitatively indicates whether the obtained calibration parameter is similar to the true value or not. In order to effectively use the obtained calibration parameter, it is important to validate the parameter through PI. Therefore, in this paper, we propose an algorithm to obtain the performance index for the calibration parameter between LiDAR and the motion sensor. This performance index is experimentally verified in various environments by Monte Carlo simulation and validated using CarMaker simulation data and real data. As a result of verification, the PI of the calibration parameter obtained through the proposed algorithm has the smallest value when the calibration parameter has a true value, and increases as an error is added to the true value. In other words, it has been proven that PI is convex to the calibration parameter. In addition, it is able to confirm that the PI obtained using the proposed algorithm provides information on the effect of the calibration parameters on mapping and localization.

## 1. Introduction

Autonomous vehicles combine information from various sensors, such as light detection and ranging (LiDAR), cameras, Global Navigation Satellite System (GNSS), and Inertial Navigation System (INS), with vehicle data to recognize the surrounding environment and determine the current vehicle status. In this case, each sensor has its own unique coordinate system. Therefore, in order to effectively integrate and use the sensor values measured based on each sensor coordinate system, it is necessary to unify each sensor coordinate system into one reference coordinate system. In order to unify the reference coordinates, it is essential to find a transformation relationship between the respective sensor coordinates and the reference coordinates. The process of obtaining such a transformation, which includes rotation and translation relationship between coordinate systems, is called extrinsic parameter calibration.

Extrinsic calibration between LiDAR and the motion sensor involves finding the transformation relationship between the LiDAR and the motion sensor. At this time, the motion sensor refers to an integrated sensor when it is integrated into one by applying a certain methodology [1] to an in-vehicle sensor, such as a yaw-rate sensor or wheel speed sensor, attached to several locations. Alternatively, the inertial measurement unit (IMU) sensor or INS sensor itself, which includes all information that can indicate vehicle movement, is defined as a motion sensor. It is extremely essential, as the result of calibration has a significant influence on the performance of LiDAR-based autonomous driving applications, such as mapping [2], localization [3,4], and object detection [5]. Therefore, research on extrinsic calibration between LiDAR and the motion sensor is being actively conducted.

Conventional LiDAR and motion sensor calibration methods include a direct calibration method that derives parameters directly using surrounding structures or calibration room and an indirect calibration method that derives parameters indirectly through an algorithm. In order to derive accurate parameters through the direct calibration method, time or human resources are greatly required. Therefore, the algorithm-based indirect calibration method is widely used. However, as this value is derived based on an algorithm, it is not always possible to derive a value similar to the actual value. Therefore, in order to effectively verify the parameters derived from in-direct calibration, it is necessary to quantitatively indicate whether this value is similar to the true value or not, and this quantitative value is called the performance index PI of the extrinsic calibration parameter.

One of the widely used methods for PI is to analyze the root mean square error (RMSE) with the ground-truth value. At this time, it is assumed that the value set when attaching the sensor or the direct calibration value previously measured is ground truth. RMSE can represent the degree of the error directly and intuitively. In real situations, however, as the measured ground truth is likely to change, it is very difficult to always obtain an accurate ground-truth value. Another method is to use standard deviation, repeatability, and convergence as performance indicators after trying indirect calibration several times. Although this method provides the characteristics of the calibration parameters, it has the disadvantage of not responding to biased results. The last method is to compare the generated map smoothness using calibration parameters to evaluate. However, this method only provides a relative PI for the calibration parameter and does not provide an intuitive and absolute PI. In addition, all three methods do not provide information on how much the derived calibration parameters affect mapping and localization.

Therefore, in order to overcome the problems of the existing performance indices, we propose an algorithm to obtain a performance index for a 6-degree-of-freedom (DoF) extrinsic calibration parameter between LiDAR and the motion sensor. The performance index obtained through this algorithm can easily derive an unbiased absolute performance index without ground truth, and it is possible to figure out how much influence it has on mapping and localization from the derived calibration parameter. The main contribution of this paper is as follows:

- It presents a novel algorithm to obtain a performance index for the 6-DoF extrinsic calibration parameter between LiDAR and the motion sensor based on localization and mapping. It can determine how much it influences the localization performance as well as the goodness of fit for extrinsic calibration results.
- It was experimentally verified through Monte Carlo simulation as to whether it is effective in various environments.
- In addition, more accurate correction parameters can be derived by applying global optimization using the obtained PI as a cost function, and this is briefly introduced in Appendix A.

The remainder of this paper is organized as follows: Section 2 briefly reviews previous studies. In Section 3, we introduce the proposed algorithm to obtain the PI of the calibration parameter. We verify the validity of the proposed algorithm in various environments through Monte Carlo simulation in Section 4. Then, we show the experimental results using simulation and real data in Section 5, and, finally, the conclusion is described in Section 6.

## 2. Previous Studies

### 2.1. Approaches for Extrinsic Parameter Calibration

The traditional method for extrinsic parameter calibration between sensors can be largely divided into direct calibration and indirect calibration. Direct calibration measures distance and angle manually by either placing landmarks to control points or by using known spatial maps, such as calibration room [6,7,8]. These methods, however, consume both time and labor with experts. To address these issues, indirect calibration approaches based on the motion data of each sensor have been studied in recent decades. They are generic approaches because they exploit relative pose information regardless of the type of sensor. There are various ways to find a solution for the calibration parameter.

Hand–eye calibration finds the relationship between the sensor on the robot’s end-effector and base frame of the robot [9,10,11]. They attempt to solve a homogeneous matrix equation formed as “AX = XB” where “A” is related to the relative pose of a reference motion sensor and “B” is related to the relative pose of a target sensor. In this problem, it is important to obtain the sensor’s trajectory by measurements because they cannot provide the sensor’s relative pose information directly.

Reference [12] estimates calibration parameters for LiDARs without any specific and known environmental features based on a fully unsupervised approach. It does not require a specific calibration target. With contiguous surface assumption, it finds globally consistent parameters by optimizing an energy function that evaluates whether points are far away from surfaces.

The graph-optimization-based approach was also proposed to consider properties for sensors mounted in an autonomous vehicle [13]. By applying additional constraints, it improved robustness and reliability.

### 2.2. Approaches for Evaluation of Extrinsic Calibration Parameter

The extrinsic calibration parameters obtained through the various methods introduced above are evaluated in terms of how appropriate they are with some evaluation indices. There are two main ways to evaluate them: with and without ground-truth data. Ground truth can be found by applying various calibration methods and further optimization techniques. For example, the KITTI dataset provides the ground-truth extrinsic calibration parameters between LiDAR-IMU that are calculated based on the hand–eye calibration [7]. The Lyft Level 5 dataset conducted manual calibration with its own calibration room [8]. In addition, ref. [14] also applied a method of measuring parameters using CAD drawings.

Several studies evaluate their calibration parameter by errors between estimated ones and ground truth that are given by either the KITTI dataset or their own dataset [7,13,15]. With ground truth, a mean and standard deviation of errors can represent the reference best fitness of the system. However, these methods cannot be used for online calibration problems that require considering environments in which parameters can be varied over time or events and are inefficient as they require a lot of time and cost when the parameters are changed.

For real data, it is difficult to find ground-truth data with simulation. In this case, the standard deviation of the parameters, convergence, or repeatability can be used to evaluate how the proposed system is robust and stable [13,16,17,18]. The mean of parameters are useful to figure out the precision of the system but cannot evaluate accuracy from the biased results. A performance index based on map smoothness that is related to mapping performance was applied to evaluate parameters [13,19,20,21]. Map smoothness is introduced as a metric that can be used to determine how blurry a map will be generated through the estimated calibration parameter. This has the advantage of being able to see how well the estimated parameters fit the mapping application. However, to be used as an absolute performance index, it is inappropriate because it is a relative indicator that cannot provide a standard value for fit.

### 2.3. Limitations of Previous Methods

The requirements to cover the limitations of the existing method are summarized as follows:

- It should not require obtaining the ground-truth parameters, which require much time and cost.
- It should have an unbiased value.
- It should have an intuitive and absolute value, not a relative indicator.

The proposed index can overcome all of them, and if it is used as a cost function that minimizes this index, it can also be used to find the globally optimal calibration parameter through global optimization techniques. In addition, unlike other indices, the proposed performance index can check how much influence the mapping and localization have on performance through the obtained performance index. The next section introduces how to obtain this evaluation index.

## 3. Calibration Performance Index of LiDAR and Motion Sensor

**Figure 1:**
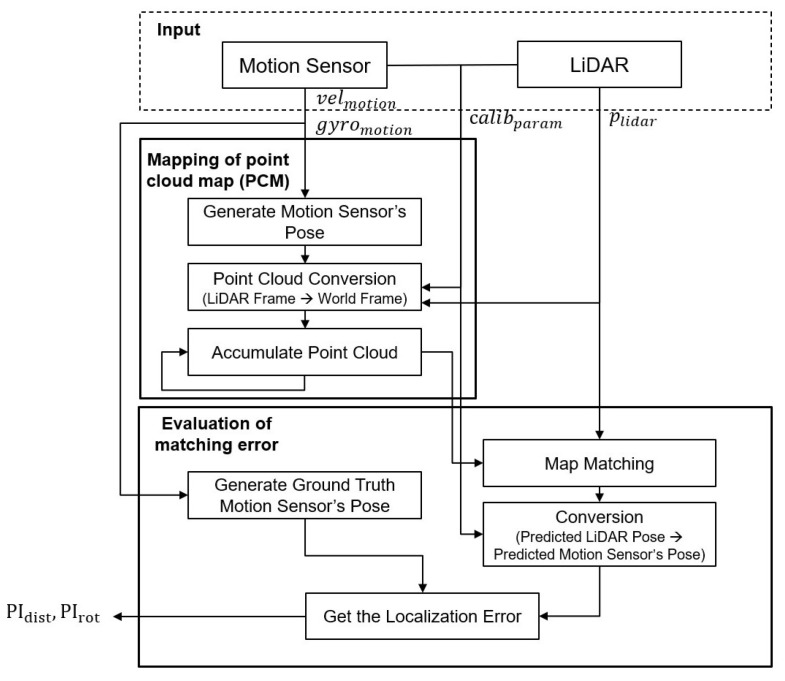
Algorithm of obtaining the proposed performance index of the calibration parameter.

This section introduces the algorithm used to obtain the performance index of the calibration parameter between LiDAR and the motion sensor. It is based on two properties that consider map smoothness, introduced in the previous study. The first principle is that the mapping result is accurate and has less blurring when point cloud mapping uses a good calibration parameter, whereas the mapping result using a bad calibration parameter is inaccurate and has large blurring. The second principle is that the matching result is good when matching between an accurate map and point cloud, whereas matching is inaccurate when matching between an inaccurate and blurred map and point cloud. To evaluate a calibration parameter using these properties, the algorithm to obtain the proposed PI consists of two steps. First, the point cloud map (PCM) is generated using motion data obtained from the motion sensor, the point cloud obtained from LiDAR, and a calibration parameter for performance evaluation. Second, the matching error between the created PCM and the input point cloud used for mapping is obtained. The obtained distance and rotation matching error is used as the performance index $PI_{dist}$, $PI_{rot}$. This algorithm is shown in Figure 1 and is described in detail in Section 3.1 and Section 3.2.

**Figure 2:**
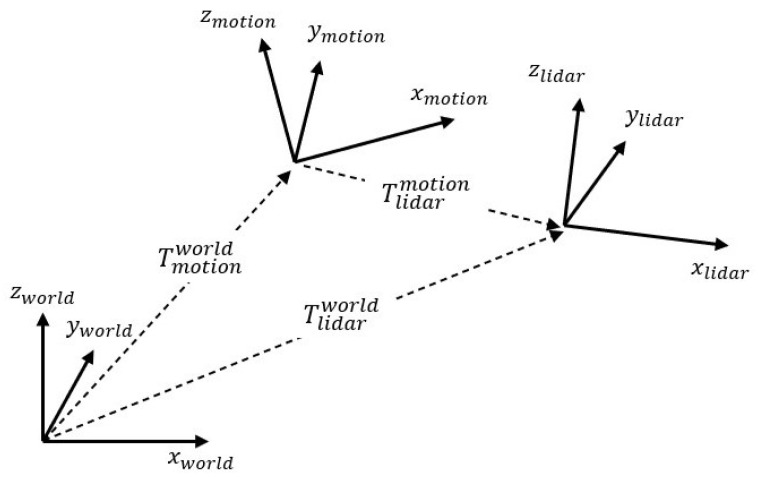
Relationship between the coordinate systems: world, motion, LiDAR coordinates.

Before the detailed algorithm, we define the notation that is used in this algorithm and describe the input data. Three coordinates: world coordinate; motion sensor coordinate; LiDAR coordinate; are used in this algorithm. The relationship between each coordinate system is as follows and is shown in Figure 2. ${T_{lidar}^{world}}_{i}$ is the transformation matrix consisting of LiDAR’s 6-DoF pose in time *i* based on world coordinate. ${T_{lidar}^{world}}_{i}\in SE\left(3\right)$ is equal to $\left[{R_{lidar}^{world}}_{i}|{t_{lidar}^{world}}_{i}\right]$, where ${R_{lidar}^{world}}_{i}\in SO\left(3\right)$ is a rotation matrix that consists of LiDAR’s orientation in time *i*, and ${t_{lidar}^{world}}_{i}\in R^{3}$ is a translation vector that consists of LiDAR’s position in time *i*. ${T_{motion}^{world}}_{i}$ is the transformation matrix consisting of the motion sensor’s 6-DoF pose in time *i* based on world coordinate. ${T_{motion}^{world}}_{i}\in SE\left(3\right)$ is equal to $\left[{R_{motion}^{world}}_{i}|{t_{motion}^{world}}_{i}\right]$, where ${R_{motion}^{world}}_{i}\in SO\left(3\right)$ is a rotation matrix that consists of the motion sensor’s orientation in time *i*, and ${t_{motion}^{world}}_{i}\in R^{3}$ is a translation vector that consists of the motion sensor’s position in time *i*. ${T_{lidar}^{motion}}_{i}$ is the calibration transformation matrix consisting of the 6-DoF calibration parameter from the motion sensor to LiDAR. The calibration parameter is a (6 × 1) vector consisting of the 3-axis translation distance ($x_{lidar}^{motion}$, $y_{lidar}^{motion}$, $z_{lidar}^{motion}$) and rotation angles, which are represented by ZYX Euler angle ($roll_{lidar}^{motion}$, $pitch_{lidar}^{motion}$, $yaw_{lidar}^{motion}$) of the LiDAR on the motion sensor. $R_{lidar}^{motion}\in SO\left(3\right)$ and $t_{lidar}^{motion}\in R^{3}$ can be created using a calibration parameter, and $T_{lidar}^{motion}\in SE\left(3\right)$ is equal to $\left[R_{lidar}^{motion}|t_{lidar}^{motion}\right]$.

The data used for point cloud mapping and evaluation using the matching error are as follows. $p_{lidar_{i}}$ is the point cloud on the LiDAR coordinate obtained from the LiDAR in time *i*. If it consists of *n* points, it is a matrix of size (*n* × 3). $gyro_{motion_{i}}$ and $vel_{motion_{i}}$ are the angular velocity and linear velocity on motion sensor coordinate obtained from the motion sensor in time *i*. It contains 3-axis angular velocities and linear velocities. Finally, $calib_{param}$ is a (6 × 1) vector including 3-axis translation distances and rotation angles between LiDAR and the motion sensor, and it is a variable for evaluating performance. A point cloud map is created using $p_{lidar_{i}}$, $gyro_{motion_{i}}$, $vel_{motion_{i}}$, and $calib_{param}$ given as inputs. Next, we evaluate matching error using this generated map and $p_{lidar_{i}}$, $gyro_{motion_{i}}$, $vel_{motion_{i}}$ and $calib_{param}$ are used to create the point cloud map. This process is described in detail below.

### 3.1. Mapping of Point Cloud Map (PCM)

It receives $p_{lidar_{i}}$, $gyro_{motion_{i}}$, $vel_{motion_{i}}$, and $calib_{param}$ as input and output PCMs on world coordinates. In general, the motion sensor is faster than the LiDAR. Therefore, when collecting data from the motion sensor quickly, it is assumed that LiDAR data with a slow frequency is collected at the same time at a certain point in time.

#### 3.1.1. Generation of Motion Sensor’s Pose Using Input Motion Data

By applying input motion data such as $gyro_{motion_{i}}$ and $vel_{motion_{i}}$ to dead reckoning or other vehicle models, it is necessary to obtain the 6-DoF motion sensor’s poses. The homogeneous transformation matrix $t_{motion}^{world}$ is obtained using these poses.

#### 3.1.2. Point Cloud Conversion

To create a point cloud map, $p_{lidar_{i}}$, is given as input of a LiDAR coordinate and is converted into the point cloud of the world coordinate. At time *i*, the point cloud $p_{lidar_{i}}$ can be converted to $p_{world_{i}}$, point cloud on a world coordinate using Equations (1) and (2). To convert point cloud using a transformation matrix, $p_{lidar_{i}}$ is converted into $p_{lidar\_hom_{i}}=\left[p_{lidar_{i}}|1\right]$, which is in the homogeneous coordinate.

(1) $${T_{lidar}^{world}}_{i}={T_{motion}^{world}}_{i}T_{lidar}^{motion}$$

(2) $${\left(p_{world\_hom_{i}}\right)}^{T}={T_{lidar}^{world}}_{i}{\left(p_{lidar\_hom_{i}}\right)}^{T}$$

#### 3.1.3. Generation of Point Cloud Map by Accumulation

**Figure 3:**
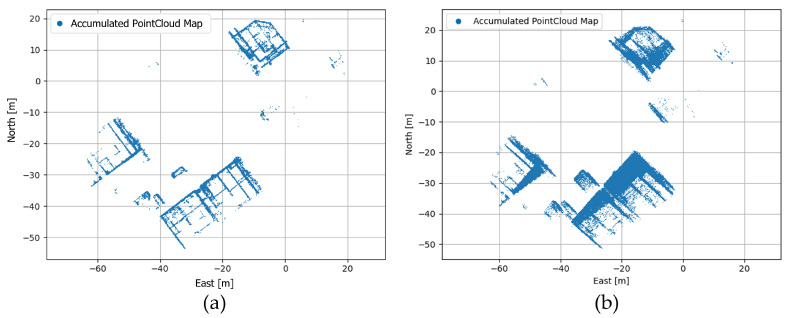
(**a**) Result of accumulated point cloud map (PCM) using the good calibration parameter; (**b**) result of accumulated PCM using the wrong calibration parameter.

By accumulating the overall point cloud on the world coordinate, a point cloud map on a world coordinate can be obtained. If the map is created using the correct calibration parameter between the motion sensor and the LiDAR, it will generate an accurate map with less blurring. On the other hand, if a map is created using the wrong calibration parameter, it will create a blurred and inaccurate map. The mapping results according to the calibration parameter are shown in Figure 3, and the algorithm is described in Algorithm 1.

(3) $$PCM=\cup_{i}p_{world_{i}}$$

**Algorithm 1** Point Cloud Mapping
1: **Inputs**
    Motion data in time *i*: $gyro_{motion_{i}}$, $vel_{motion_{i}}$
    Point cloud in time *i*: $p_{lidar_{i}}$
    Calibration transformation matrix: $T_{lidar}^{motion}$
2: **Output**
    Accumulated point cloud map: PCM
3: **for** *i* = 1 to *N* **do**
4:    ${T_{motion}^{world}}_{i}\leftarrow VehicleModel\left(gyro_{motion_{i}},vel_{motion_{i}}\right)$
5:    ${T_{lidar}^{world}}_{i}\leftarrow {T_{motion}^{world}}_{i}T_{lidar}^{motion}$
6:    $p_{world_{i}}\leftarrow {T_{lidar}^{world}}_{i}p_{lidar_{i}}$
7:    $PCM\leftarrow Accum(PCM,p_{world_{i}})$
8: **end for**


### 3.2. Evaluation of Matching Error

To evaluate matching error, it receives created PCM and $p_{lidar}$, $gyro_{motion}$, $vel_{motion}$, $calib_{param}$ used to make PCM as inputs, and outputs the matching error. The assumptions about time synchronization are the same as in Section 3.1.

#### 3.2.1. Generation of Ground-Truth Motion Sensor’s Pose Using Input Motion Data

The 6-DoF motion sensor’s poses and transformation matrix $T_{motion}^{world}$ can be obtained using the same method and data as when generating PCM.

#### 3.2.2. Generation of Predicted Motion Sensor’s Pose Using Point Cloud-PCM Matching

The 6-DoF-predicted LiDAR poses on the world coordinate are obtained through matching between the created PCM on the world coordinate and the point cloud on the LiDAR coordinate given as input. In this case, matching is performed using a registration method such as point-to-point, point-to-line, point-to-plane, generalized ICP, and NDT [22,23,24,25,26], and the matching result becomes the pose of the LiDAR in the world coordinate. Therefore, a value near the LiDAR pose is required as the initial value of registration. If the LiDAR pose used for mapping is given as an initial value in registration, the value applied to the initial value on the input point cloud will almost coincide with the mapping point. Therefore, this initial value will be the registration result. In this case, it does not satisfy the property that the matching result is not accurate when matching between the blurred map and the point cloud. Therefore, the property is satisfied by not using the LiDAR pose as the initial value of registration, but using the value added with the random value to the LiDAR pose as the initial value. The random value to be used for the initial value at time *i* consists of $x_{rand_{i}}$, $y_{rand_{i}}$, $z_{rand_{i}}$, $roll_{rand_{i}}$, and $pitch_{rand_{i}}$, $yaw_{rand_{i}}$, and $T_{rand_{i}}\in SE\left(3\right)$, a homogeneous transformation matrix, can be obtained through these parameters. At this time, these $x_{rand_{i}}$, $y_{rand_{i}}$, $z_{rand_{i}}$, $roll_{rand_{i}}$, $pitch_{rand_{i}}$, and $yaw_{rand_{i}}$ values are obtained by extracting random values within a reasonable value of the maximum error that a calibration parameter can have. After obtaining the random value, the initial value of registration at time *i*, $T_{init_{i}}\in SE\left(3\right)$ can be obtained through Equation (4).

(4) $$T_{init_{i}}={T_{lidar}^{world}}_{i}T_{rand_{i}}$$

**Figure 4:**
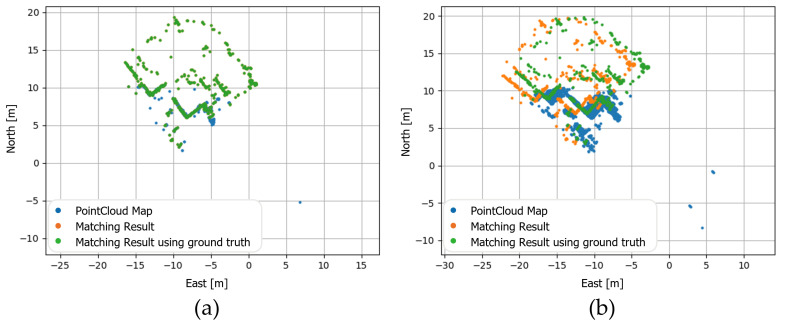
(**a**) Result of matching between PCM and point cloud using the good calibration parameter; (**b**) result of matching using the wrong calibration parameter; blue points represent PCM on world coordinate, orange points represent matched points using registration, and green points represent points that are converted using ground truth LiDAR pose.

The predicted LiDAR pose on the world coordinate obtained through matching between the PCM and the point cloud in time *i* is defined as ${T_{pred\_lidar}^{world}}_{i}$. When matching using sharp and accurate map, ${T_{pred\_lidar}^{world}}_{i}$ will be obtained, which is similar to the LiDAR pose used when generating the map, ${T_{lidar}^{world}}_{i}$. On the other hand, when matching using blurred and inaccurate map, ${T_{pred\_lidar}^{world}}_{i}$ will be far from the LiDAR pose used to generate the map. The matching results according to the calibration parameter are shown in Figure 4.

The predicted motion sensor’s pose in the world coordinate can be obtained by multiplying the derived predicted LiDAR pose in the world coordinate, ${T_{pred\_lidar}^{world}}_{i}$, by the calibration parameter inversely. The predicted motion sensor’s pose in time *i* is defined as ${T_{pred\_motion}^{world}}_{i}$, and it is expressed as the following equation:

(5) $${T_{pred\_motion}^{world}}_{i}={T_{pred\_lidar}^{world}}_{i}{\left(T_{lidar}^{motion}\right)}^{-1}$$

Similarly, the predicted motion sensor’s pose obtained using the correct calibration parameter will almost match the ground truth motion sensor’s pose which is obtained by applying motion data to dead reckoning or other vehicle models, and the predicted motion sensor’s pose obtained using the incorrect calibration parameter will be far from the ground truth pose.

#### 3.2.3. Obtaining the Localization Error between Ground-Truth Motion Sensor’s Pose and Predicted Pose

Localization errors between ${T_{pred\_motion}^{world}}_{i}$ and ${T_{motion}^{world}}_{i}$ are calculated, and the distance root mean square error (RMSE) and rotation RMSE from these distance and rotation errors, obtained from the entire data, can be derived. The distance RMSE and rotation RMSE are defined as $PI_{dist}$, $PI_{rot}$, respectively. At this time, the amount of computation can be reduced by obtaining PI only at the time selected through distance sampling instead of obtaining PI for the entire data. As a result, when the correct calibration parameter is used in this algorithm, $PI_{dist}$ and $PI_{rot}$ will be small, and the larger the error in the calibration parameter, the larger $PI_{dist}$, $PI_{rot}$ will be. This is proven through Monte Carlo simulation in Section 4.
**Algorithm 2** Evaluation of Matching Error  **Inputs**     Motion data in time *i*: $gyro_{motion_{i}}$, $vel_{motion_{i}}$     Point cloud in time *i*: $p_{lidar_{i}}$     Calibration transformation matrix: $T_{lidar}^{motion}$     Accumulated point cloud map: PCM2:  **Output**     Performance index according to calibration parameter: $PI_{dist},PI_{rot}$  **for** *i* = 1 to *N* **do**4:    ${T_{motion}^{world}}_{i}\leftarrow VehicleModel\left(gyro_{motion_{i}},vel_{motion_{i}}\right)$    ${T_{lidar}^{world}}_{i}\leftarrow {T_{motion}^{world}}_{i}T_{lidar}^{motion}$6:    ${T_{rand}}_{i}\leftarrow RandomGenerator$    ${T_{init}}_{i}\leftarrow T_{lidar}^{world}T_{rand_{i}}$8:    ${T_{pred\_lidar}}_{i}\leftarrow MatchingbtwPCMandPC\left(PCM,p_{lidar_{i}},T_{init_{i}}\right)$    ${T_{pred\_motion}}_{i}\leftarrow T_{pred\_lidar_{i}}{\left(T_{lidar}^{motion}\right)}^{-1}$10:   $dist\_err_{i}\leftarrow t_{pred\_motion_{i}}-t_{motion_{i}}$     $rot\_err_{i}\leftarrow R_{pred\_motion_{i}}⊖R_{motion_{i}}$12: **end for**   $PI_{dist}\leftarrow RMSE\left(dist\_err\right)$14: $PI_{rot}\leftarrow RMSE\left(rot\_err\right)$

## 4. Experimental Verification Based on Monte Carlo Simulation

In this section, it was experimentally verified that the proposed PI is convex for the added calibration parameter error using the Monte Carlo simulation method. It is proved that if the error of the calibration parameter is 0, the proposed PI will be 0, and the proposed PI increases when the calibration parameter is added. After introducing the simulation environment, variables, and equations for verification, this verification process and results will be explained.

### 4.1. Environment of Verification

#### 4.1.1. Definition of Variables

**Figure 5:**
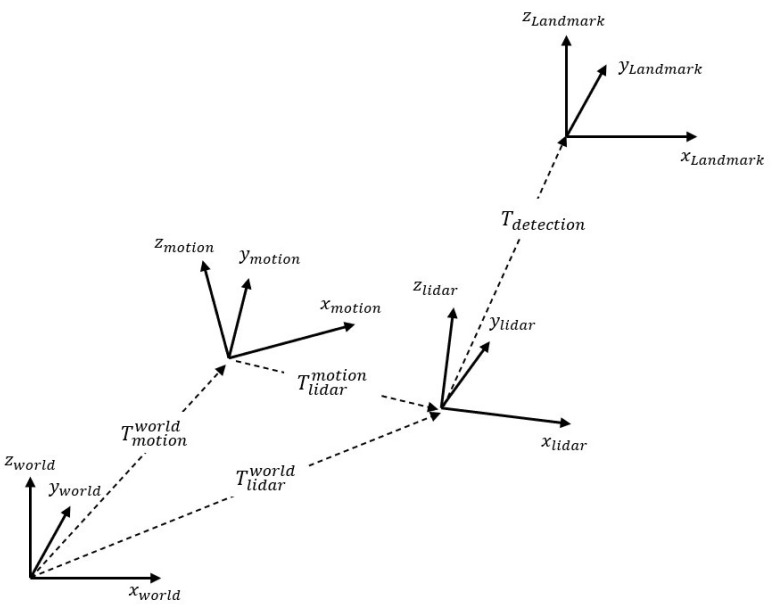
Relationship between the coordinate systems: world, motion, LiDAR, landmark coordinates.

The variables used for validation are as shown in Figure 5. $T_{motion}^{world}$, which is a transformation matrix composed of 6-DoF pose of motion sensor, and $T_{motion}^{lidar}$ composed of a calibration parameter are obtained through Monte Carlo sampling. The 6-DoF pose of LiDAR can be obtained through Equation (1). In order to exclude registration error during PCM-point cloud matching, it is assumed that there is a directional landmark, and $T_{landmark}$ is the transformation matrix of this landmark. Through this landmark, the transformation matrix $T_{detection}$, which is the detection of the landmark from the LiDAR, is obtained from Equation (6). Figure 5 shows the relationship described above.

(6) $$T_{detection}=T_{landmark}^{world}{\left(T_{lidar}^{world}\right)}^{-1}$$

#### 4.1.2. Generation of Motion Sensor’s Pose and Calibration Parameter through Monte Carlo Sampling

**Figure 6:**
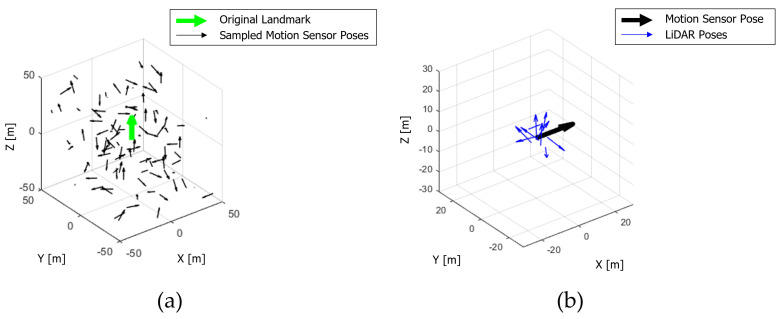
(**a**) Landmark for detection (green) and 100 sampled motion sensor’s poses (black). (**b**) 10 sampled LiDAR poses (blue) based on one motion sensor’s pose (black).

Using Monte Carlo simulation, we generate *n* different motion sensor’s poses and *m* different calibration parameters. Assume that a directional landmark’s x, y, and z positions are each 0, and the x-direction of the coordinate points upward. Motion sensor poses with random x, y, and z positions within a radius of 50 m of this landmark, and random roll, pitch, and yaw orientations within 360 degrees, are generated. The landmark and the generated *n* motion sensor’s poses are shown in Figure 6.

### 4.2. Process of Verification Using Monte Carlo Simulation

#### 4.2.1. Generation of Landmark Using *n* Motion Sensor’s Pose and One Calibration Parameter

**Figure 7:**
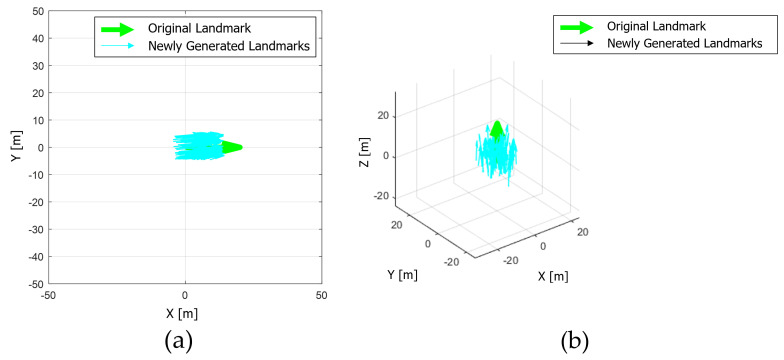
(**a**,**b**) Landmark for detection (green) and newly generated blurred landmark using Equation (8) (cyan).

New landmarks are generated using one calibration parameter and *n* motion sensor’s poses. At this time, the process of generating new landmarks using the *i*th motion sensor’s pose and the transformation of detection between LiDAR and landmark is expressed in Equation (7). As the calibration parameter does not contain an error, newly generated landmarks match the actual landmark. However, the calibration parameter to be evaluated contains errors. Therefore, newly generated landmarks obtained using the calibration parameter, including the calibration error, can be derived using Equation (8), and it is shown in Figure 7.

(7) $$T_{new\_landmark_{i}}={T_{motion}^{world}}_{i}T_{lidar}^{motion}T_{detection_{i}}$$

(8) $$T_{new\_landmark_{i}}={T_{motion}^{world}}_{i}T_{lidar}^{motion}T_{error}T_{detection_{i}}$$

To obtain PI, it is necessary to obtain the predicted LiDAR pose through matching to obtain the predicted motion sensor’s pose described in Section 3.2. Unlike previously described, to exclude registration errors during matching, one must randomly select one of the newly generated landmarks instead of registration. If the index of the randomly selected landmark is defined as *j*, then the selected landmark is expressed as Equation (9). The *i*th predicted LiDAR pose using this landmark as a matching result can be obtained through Equation (10). The predicted motion sensor’s pose can be obtained by inversely multiplying the predicted LiDAR pose by the calibration parameter containing the error. The *i*th predicted motion sensor’s pose is expressed in Equation (11).

(9) $${T_{sel\_landmark}^{world}}_{i}={\left({T_{motion}^{world}}_{j}T_{lidar}^{motion}T_{error}T_{detection_{j}}\right)}_{j=rand(1,...,n)}$$

(10) $${T_{pred\_lidar}^{world}}_{i}={\left({T_{motion}^{world}}_{j}T_{lidar}^{motion}T_{error}T_{detection_{j}}\right)}_{j=rand(1,\ldots ,n)}{\left(T_{detection_{i}}\right)}^{-1}$$

(11) $${T_{pred\_motion}^{world}}_{i}=T_{sel\_landmark_{i}}{\left(T_{detection_{i}}\right)}^{-1}{\left(T_{error}\right)}^{-1}{\left(T_{lidar}^{motion}\right)}^{-1}$$

Distance error can be obtained from the x, y, z component of ${T_{pred\_motion}^{world}}_{i}$, which is the transformation matrix of the predicted motion sensor’s pose, and the x, y, z component of ${T_{motion}^{world}}_{i}$ in index *i*. In addition, the rotation error is obtained from the roll, pitch, and yaw components of ${T_{pred\_motion}^{world}}_{i}$ and ${T_{motion}^{world}}_{i}$. The distance and rotation errors are calculated from the indexes from 1 to *n*, respectively, and distance RMSE and rotation RMSE are obtained through these. This distance, rotation RMSE is used as $PI_{dist},PI_{P}rot$ according to the calibration parameters. To verify that $PI_{dist}$ and $PI_{rot}$ increase as the calibration error increases, the calibration error is added, as shown in Table 1.

#### 4.2.2. Generation of Landmark Using *n* Motion Sensor’s Pose and *m* Calibration Parameter

The above procedure was performed for *m* calibration parameters. Therefore, Monte Carlo simulation was performed by adding each error in Table 1 to the total number of cases of *n* × *m*.

### 4.3. Result of Verification

**Figure 8:**
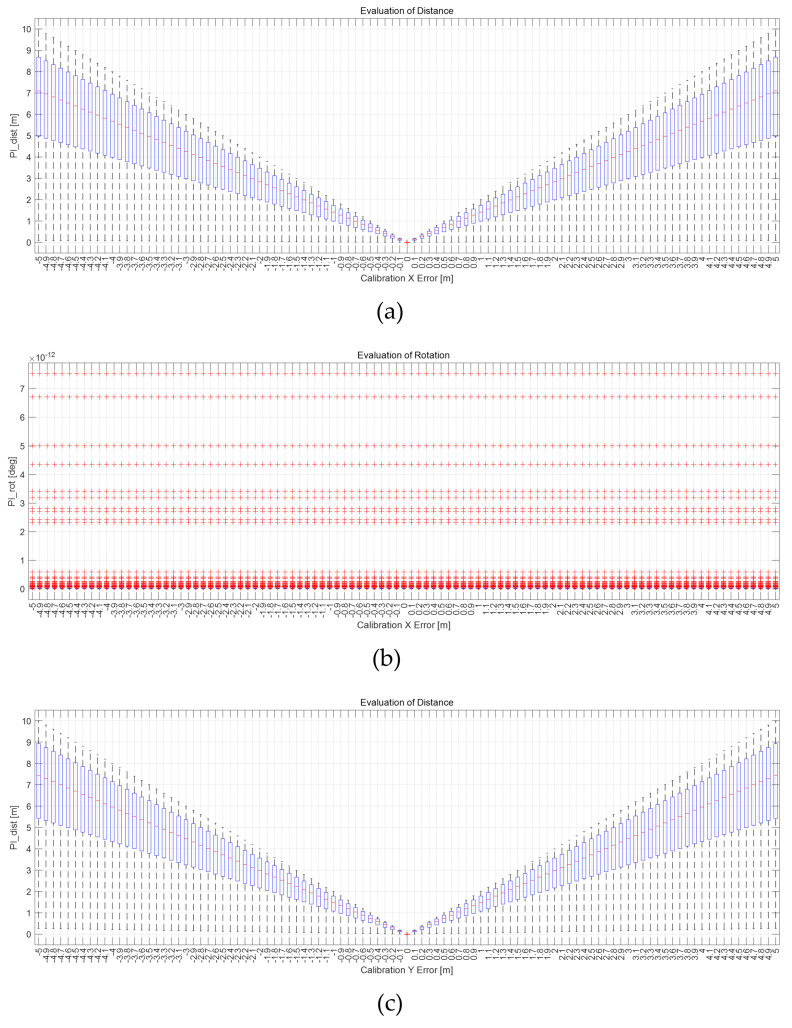

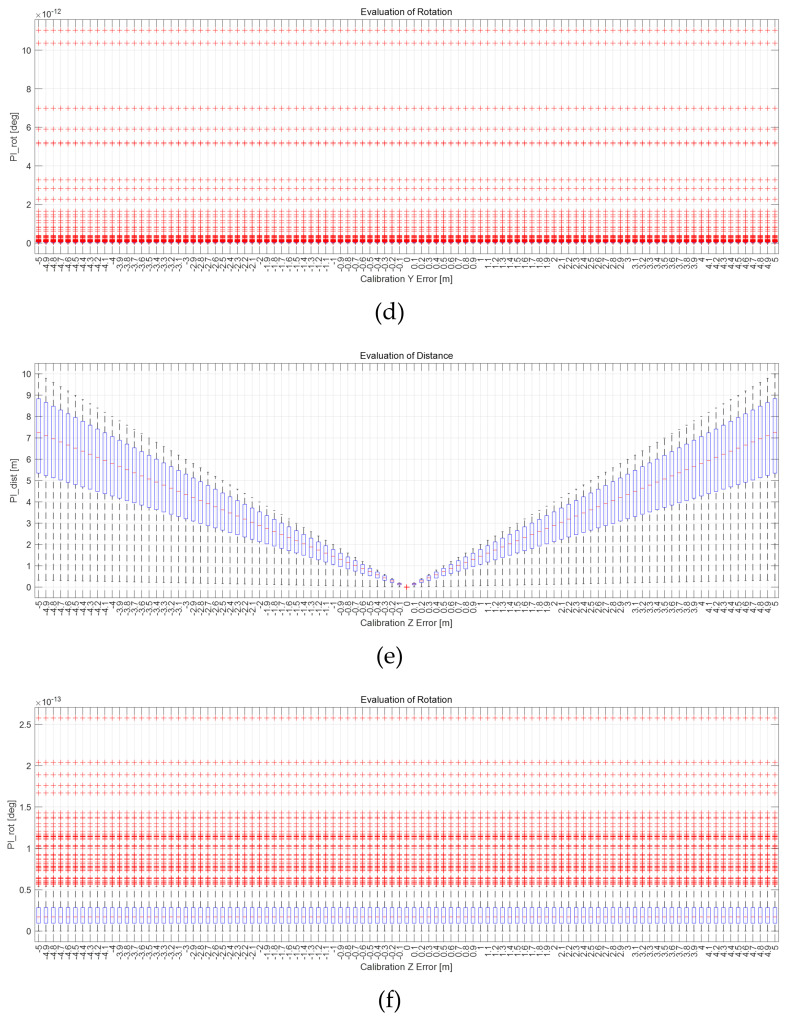

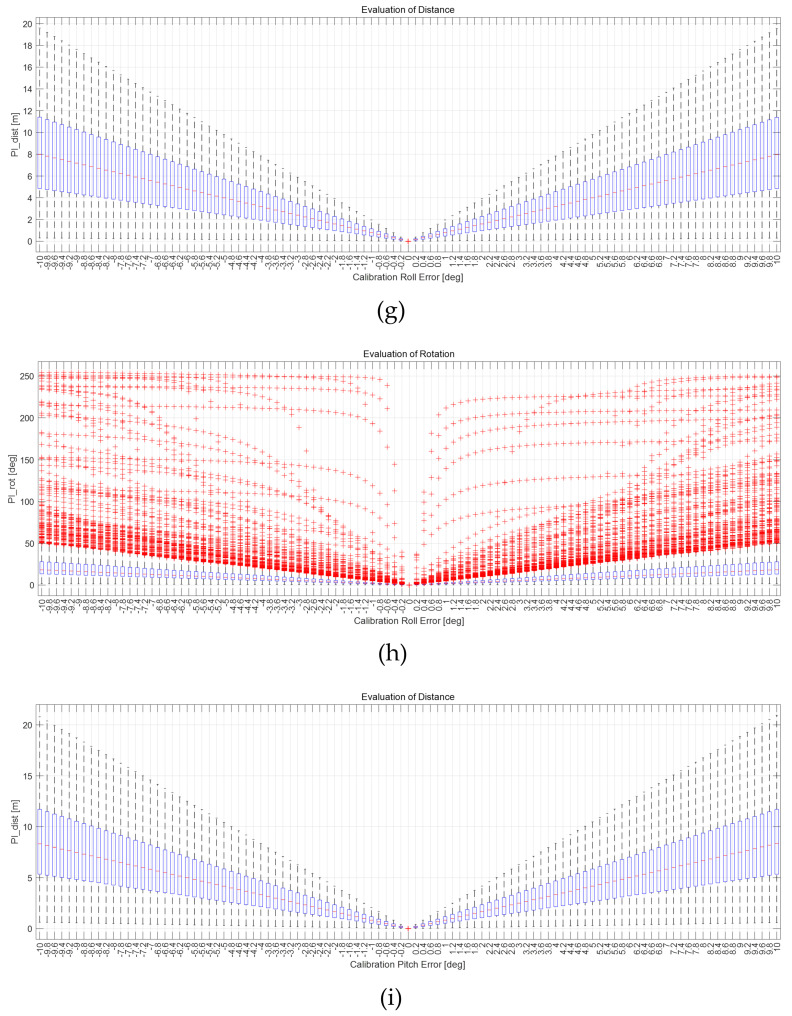

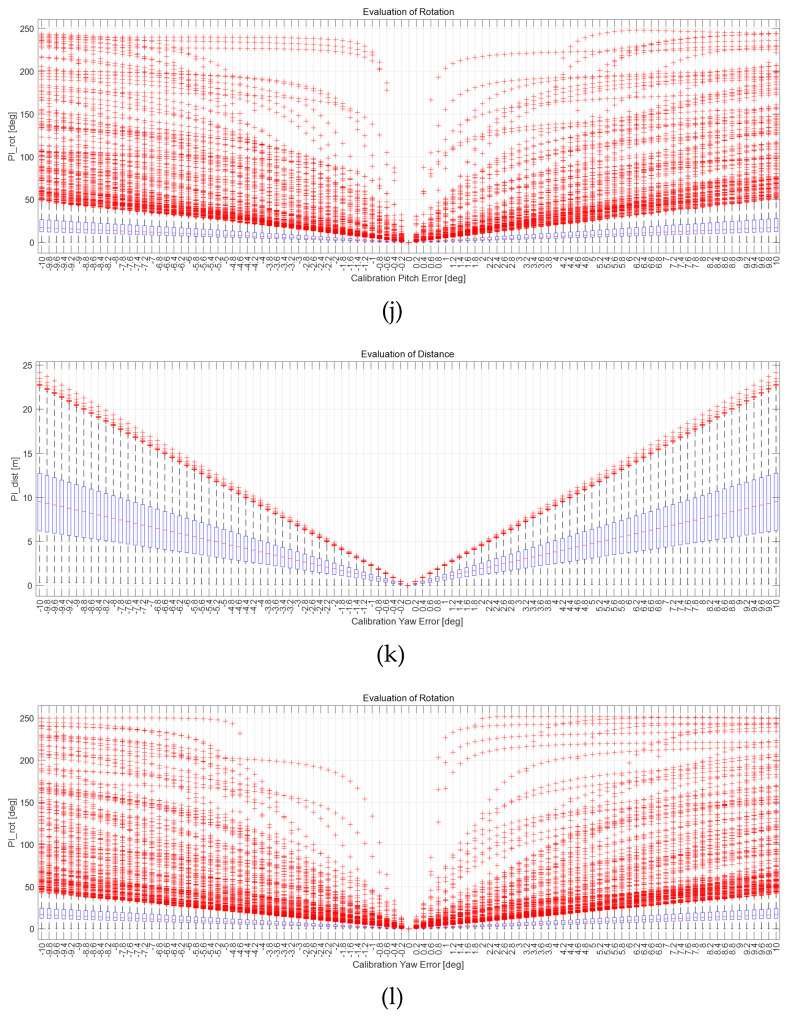
(**a**,**c**,**e**,**g**,**i**,**k**) are $PI_{dist}$ when adding each x, y, z, roll, pitch, and yaw error to the calibration parameter as much as in Table 1. (**b**,**d**,**f**,**h**,**j**,**l**) are $PI_{rot}$ when adding error.

The results for *n* × *m* cases are shown in Figure 8 as box plots. The x-axis of the graph represents the calibration parameter error, and the y-axis represents $PI_{dist}$ or $PI_{rot}$ obtained through Monte Carlo simulation. The error is 0 at the center of the x-axis and increases as it goes on both sides in the x-axis, as in Table 1. The result for *n* × *m* cases for each error are shown as box plots. It can be confirmed that the red line, which is the average of the entire simulation, is 0 when the error is 0, and when the error increases, the average value also increases. However, if there is no rotation error in the calibration parameter, $T_{pred\_motion}^{world}$ and $T_{motion}^{world}$, which are the rotation matrix of $T_{pred\_motion}^{world}$ and $T_{motion}^{world}$, are equal by Equation (11). Therefore, in this case, $PI_{rot}$ becomes 0. Through Monte Carlo simulation, it was proved that the proposed PI is convex according to the calibration error, except the $PI_{rot}$ when only x, y, and z errors are included. Therefore, the proposed PI is valid for various motion sensor movements and calibration parameters.

## 5. Experiment

In this section, the validity of the proposed algorithm was verified using simulation data and real data. Experiments using simulation data are focused on verifying whether the algorithm is valid in various environments. Experiments using real data are focused on verifying that the algorithm is valid in a noisy real environment.

### 5.1. Experiment Using Simulation Data

#### 5.1.1. Simulation Environment

The purpose of the experiments is to verify the validity of the proposed performance index, and it was evaluated using simulation data in various environments. Various environments consist of the various movements of sensors and calibration configuration between LiDAR and the motion sensor. Therefore, it was verified using IPG-CarMaker, the virtual test drive simulator that can generate sensor configurations and a sensor’s movements as desired by the user. Data were generated from one motion sensor at 10 Hz, and six time-synchronized Velodyne vlp-16 LiDARs. The configuration of various calibration parameters used in the experiment is shown in Table 2. To verify the algorithm according to the various movements of the sensor, the trajectory of the motion sensor and the surrounding environment were set as shown in Table 3.

#### 5.1.2. Result and Analysis of Experiments

The experiment was conducted using the point-to-point ICP registration method. To reduce the effect of the initial registration value, the experiment was performed 10 times for each error, and the total PI is defined as the average value of 10 PIs. The result of the experiment using simulation data with the calibration parameter error configuration described in Table 4 is shown in Table 5.

In each scenario, $PI_{dist}$ and $PI_{rot}$ are shown when errors are added to the x, y, and z elements and to the roll, pitch, and yaw elements, respectively. In Section 4.3, it was proved that there is no change in $PI_{rot}$ when there is an error only in the x, y, and yaw components among the calibration parameters. However, it was confirmed that rotation error occurred because registration was used rather than randomly selected during matching. In addition, as there is no rotational movement of the motion sensor and LiDAR in the straight scenario, the point cloud map is not blurred, and only bias is generated. Therefore, because the basic concept of the proposed algorithm is not satisfied, there is no change in the results of $PI_{dist}$ and $PI_{rot}$. Except for this case, it was confirmed that $PI_{dist}$ and $PI_{rot}$ are convex to calibration error for all calibration parameter configurations in the remaining scenarios.

### 5.2. Experiment Using Real Data

#### 5.2.1. Experimental Environment

**Figure 9:**
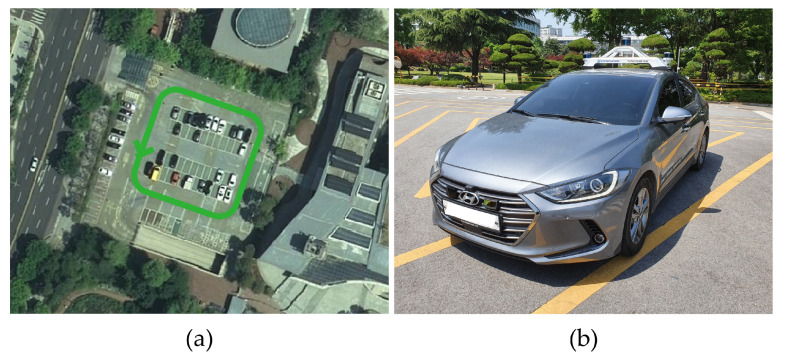
(**a**) The outdoor parking lot used for data acquisition; green line represents trajectory of the vehicle. (**b**) Autonomous vehicle platform of Konkuk University used for data acquisition.

To verify that the algorithm is valid in a noisy real environment, data was obtained from the outdoor parking lot with the autonomous vehicle platform of Konkuk University in Figure 9. This vehicle was equipped with two RoboSense rs-lidar-16 LiDARs, one RoboSense rs-lidar-32 LiDAR, and one NovAtel CPT7 INS including HG4930 MEMS IMU. At this time, the INS sensor itself was used as a motion sensor. The performance of the algorithm was verified with respect to the calibration parameters between the NovAtel CPT7 INS and the three LiDARs. The calibration parameter configuration of an INS and three LiDARs is described in Table 6. This value was acquired by obtaining the result of hand–eye calibration, which is one of the calibration methods. In addition, in order to check the correlation between pi and localization, localization RMSE was derived by implementing extended Kalman filter-based map-matching localization [27,28,29] within the same conditions.

#### 5.2.2. Results and Analysis of Experiment

The experiment was conducted using the point-to-point ICP registration method and performed 10 times for each error described in Table 4. The result of PIs using real data is shown in Table 7 by solid lines. Distance RMSE and rotation RMSE are indicated by dotted lines. As a result of the experiment using real data, it was confirmed that PIs generally had a large value because noisy real data, which degrades the performance of registration, was used. Nevertheless, it can be seen that the derived PIs are convex for the calibration parameter error. Although the localization result changed according to the tuning value setting of the localization algorithm, it showed the same distribution as the PI, regardless of the tuning value. In addition, in most cases, it was confirmed that the localization result showed a value smaller than the PI. Through this, it was possible to check the validity of the proposed PI in the real environment, which has the advantage of knowing how much the calibration parameter affects the map-matching localization.

## 6. Conclusions

This paper proposes the algorithm involving obtaining the performance index for extrinsic calibration parameters of LiDAR and the motion sensor. It was experimentally verified through Monte Carlo simulation, and its validity has been verified through experiments using CarMaker simulation data and real data. As a result, it was verified that is effective in situations except for the straight scenario without rotation movement of the motion sensor. Through these PIs, the validity of indirect calibration can be derived more easily and conveniently, and an unbiased absolute performance index can be derived. In addition, it can be known how much these PIs affect localization performance, and the more accurate calibration parameters can be obtained by applying the derived PIs to global optimization (described in Appendix A).

The algorithm for obtaining the performance index proposed in this paper can be applied to online calibration. Therefore, in the future, the study can be expanded to update the calibration parameter using this performance index, which is used as an index to determine the fault of the calibration parameter in real time.

## Figures and Tables

**Table 1:** Calibration parameter error configuration for Monte Carlo simulation.

Calibration Error Parameter	Range [m]/[deg]	Interval [m]/[deg]
x, y, z	−5∼5	0.01
roll, pitch, yaw	−10∼10	0.02

**Table 2:** Calibration parameter between one motion sensor and six LiDARs.

Sensors	Translation—x, y, z [m]	Rotation—Roll, Pitch, Yaw [deg]
Motion sensor—LiDAR1	0.0, 0.0, 0.0	0.0, 0.0, 0.0
Motion sensor—LiDAR2	3.0, 0.0, 0.0	0.0, −5.0, 0.0
Motion sensor—LiDAR3	0.8, 0.8, 1.0	−5.0, 10.0, 20.0
Motion sensor—LiDAR4	0.8, −0.8, 1.0	5.0, 10.0, −20.0
Motion sensor—LiDAR5	−0.8, 0.8, 0.0	0.0, 5.0, 150.0
Motion sensor—LiDAR6	−0.8, -0.8, 0.0	0.0, 5.0, −150.0

**Table 3:** Various environments for experiments using simulation data. Green lines represent trajectories of vehicle. Rectangular boxes represent buildings from which point cloud can be obtained.

Straight	Corner	Rectangular
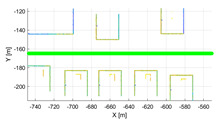	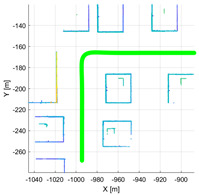	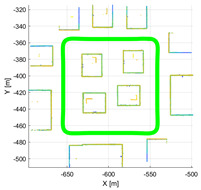
Circle	Sinuous	
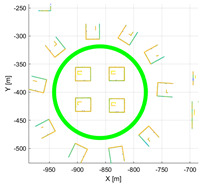	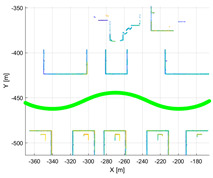	

**Table 4:** Calibration parameter error configuration.

Calibration Error Parameter	Range [m]/[deg]	Interval [m]/[deg]
x, y, z	−1∼1	0.1
roll, pitch, yaw	−5∼5	0.2

**Table 5:** Result of experiment using simulation data.

Scenario	${\mathrm{PI}}_{\mathrm{dist}}$ [m]	${\mathrm{PI}}_{\mathrm{rot}}$ [deg]
Straight	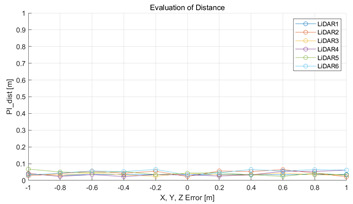	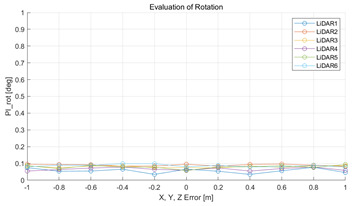
Straight	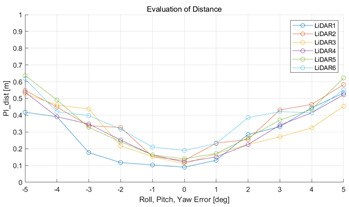	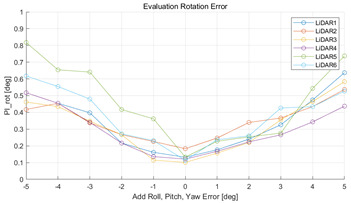
Corner	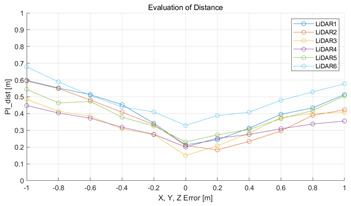	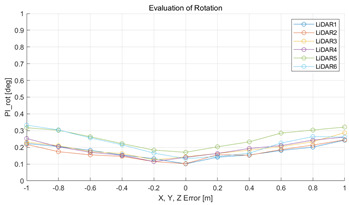
Corner	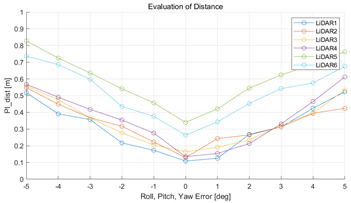	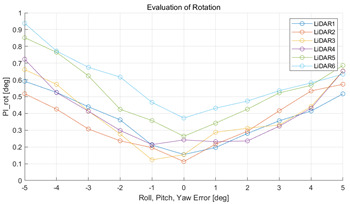
Rectangle	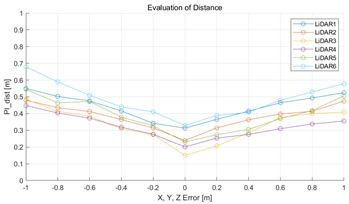	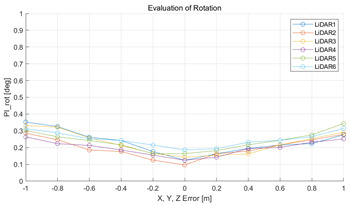
Rectangle	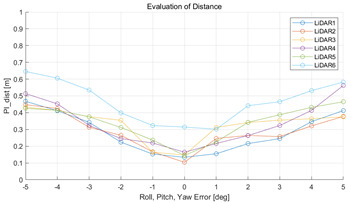	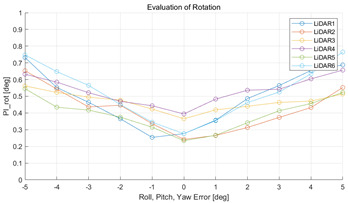
Circle	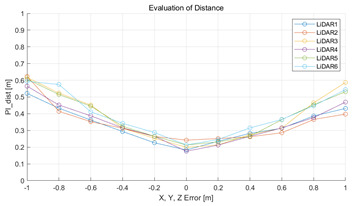	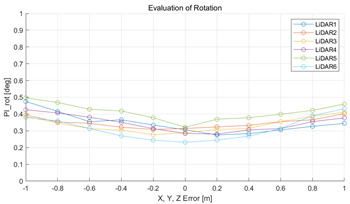
Circle	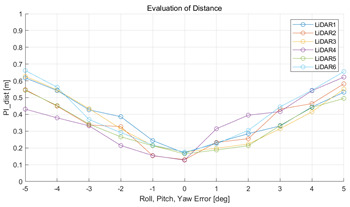	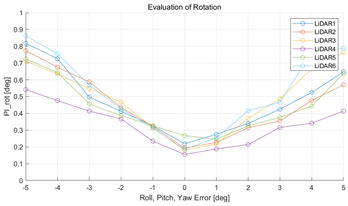
Sinuous	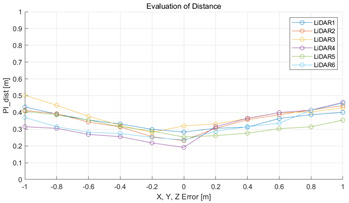	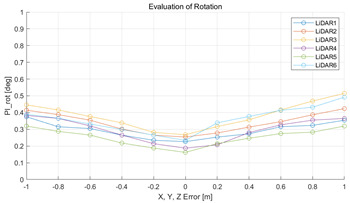
Sinuous	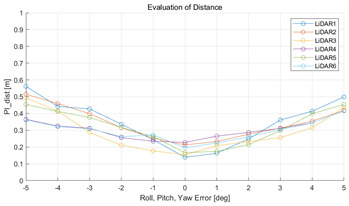	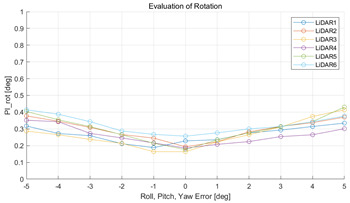

**Table 6:** Calibration parameter between one motion sensor (IMU) and three LiDARs (LiDAR1: Top-left LiDAR, LiDAR2: Top-mid LiDAR, LiDAR3: Top-right LiDAR) on the the vehicle.

Sensors	Translation—x, y, z [m]	Rotation—Roll, Pitch, Yaw [deg]
Motion Sensor—LiDAR1	0.8, 0.66, 1.51	3.0, −3.0, 0.0
Motion Sensor—LiDAR2	0.8, 0.02, 1.75	0.0, −3.0, 0.0
Motion Sensor—LiDAR3	0.8, −0.58, 1.54	0.0, −3.0, 0.0

**Table 7:** Result of experiement using real data.

${\mathrm{PI}}_{\mathrm{dist}}$ and Localization Distance RMSE [m]	${\mathrm{PI}}_{\mathrm{rot}}$ and Localization Rotation RMSE [deg]
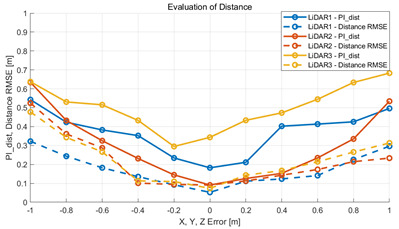	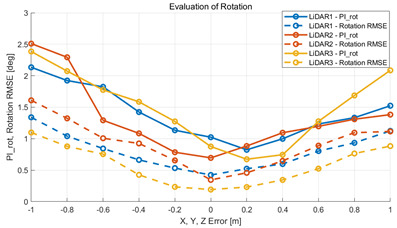
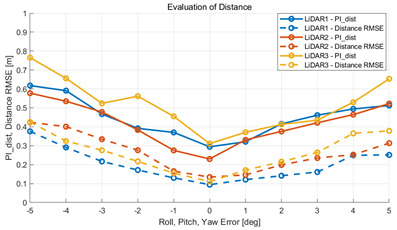	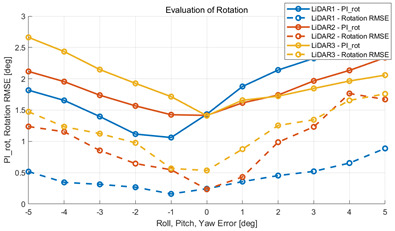

## Data Availability

Data are available upon reasonable request to the corresponding author.

## Appendix A

In this section, the process of deriving the more accurate calibration parameter through the derived PI is briefly introduced. The algorithm proposed in this paper receives calib_param, point cloud, and motion data and outputs $PI_{dist}$ and $PI_{rot}$. At this time, the values of PIs change according to calib_param. Therefore, by performing global optimization using these $PI_{dist}\left(calib\_param\right)$ and $PI_{rot}\left(calib\_param\right)$ as cost functions, $calib\_param^{*}$ that minimizes the cost function can be derived.

(A1) $$\min_{calib\_param^{*}}PI_{dist}\left(calib\_param\right)$$

(A2) $$\min_{calib\_param^{*}}PI_{rot}\left(calib\_param\right)$$
